# The influence of *UGT1A1* polymorphisms on modified FOLFIRINOX dose in double-variant-type patients with advanced pancreatic cancer

**DOI:** 10.1007/s10147-022-02186-w

**Published:** 2022-05-30

**Authors:** Tomoyuki Satake, Chigusa Morizane, Yuta Maruki, Akihiro Ohba, Yoshikuni Nagashio, Shunsuke Kondo, Susumu Hijioka, Hideki Ueno, Takuji Okusaka

**Affiliations:** Department of Hepatobiliary and Pancreatic Oncology, National Cancer Center Hospital, 5-1-1 Tsukiji, Chuo-ku, Tokyo, 104-0045 Japan

**Keywords:** *UGT1A1* polymorphisms, FOLFIRINOX, Irinotecan, Pancreatic cancer

## Abstract

**Background:**

*UGT1A1* polymorphisms should be considered when using irinotecan-containing regimens, especially in patients with a double-variant-type (DV), including homozygous for UGT1A1*28 and UGT1A1*6 and heterozygous for both UGT1A1*28 and UGT1A1*6. We investigated the safety and efficacy of modified FOLFIRINOX (mFOLFIRINOX) (irinotecan 80 mg/m^2^) in patients having DV.

**Methods:**

Patients with advanced pancreatic cancer who had received FOLFIRINOX between January 2015 and December 2019 were included in this study. Non-DV patients received the standard mFOLFIRINOX (irinotecan 150 mg/m^2^) as first-line (*non*-DV1) or second-line therapy (*non*-DV2); however, DV patients received mFOLFIRINOX (irinotecan 80 mg/m^2^) as the second-line therapy (DV2). We retrospectively evaluated the safety and efficacy of the lowered irinotecan dose in the DV2 group relative to the *non*-DV1 (safety) or *non*-DV2 (safety and efficacy) groups.

**Results:**

A total of 235 patients were eligible for this study with 118 patients in the *non*-DV1, 106 in the *non*-DV2, and 11 in the DV2 groups. Major grade 3–4 adverse events were neutropenia (33.9, 31.1, and 18.2%) and febrile neutropenia (6.8, 3.8, and 9.1%) in the *non*-DV1, *non*-DV2, and DV2 groups, respectively. The median progression-free survival was 3.4 months in the *non*-DV2 group, and 4.4 months in the DV2 group. The overall survival from the date of starting second-line chemotherapy was 8.8 months in the *non*-DV2 group and 11.5 months in the DV2 group.

**Conclusions:**

Based on our findings, the safety and efficacy of mFOLFIRINOX (irinotecan 80 mg/m^2^) in DV patients were comparable with the standard mFOLFIRINOX (irinotecan 150 mg/m^2^) in *non*-DV patients.

## Introduction

According to data from the World Health Organization (WHO), in 2020, pancreatic ductal adenocarcinoma (PDAC) was the twelfth most common cancer (495,773 incident cases) and the seventh leading cause of cancer-related deaths (466,003 deaths) [1]. In Japan, PDAC is the fourth leading cause of cancer-related death, and the mortality rate is increasing annually [2]. PDAC is expected to be the second leading cause of cancer-related deaths in developed countries in the next few years [3, 4].

PDAC has a poor prognosis, and the US “Cancer Statistics, 2019” suggests the lowest five-year relative survival rate for PDAC at 9% for all stages and only 3% for metastatic disease (the most common form) [4]. In metastatic or locally advanced PDAC, patients cannot undergo surgery; therefore, systemic therapy can be used to prolong life expectancy and improve (or preserve) quality of life.

With the results of the PRODIGE 4/ACCORD 11 trial, FOLFIRINOX (every two weeks: oxaliplatin 85 mg/m^2^ d1, irinotecan 180 mg/m^2^, leucovorin 400 mg/m^2^, 5-fluorouracil (5-FU) 400 mg/m^2^, and 5-fluorouracil 2400 mg/m^2^ over 46 h) has become a first-line therapeutic option for metastatic PDAC [5]. FOLFIRINOX offers a survival benefit over gemcitabine monotherapy [median overall survival (OS) 11.1 months vs. 6.8 months, *P* < 0.001] but with high rates of grade 3–4 adverse events, such as neutropenia, febrile neutropenia, thrombocytopenia, diarrhea, and sensory neuropathy.

In clinical practice, many oncologists reduce the dose of one or more of the FOLFIRINOX components (5-FU, oxaliplatin, or irinotecan) to minimize toxicity. Ozaka et al. reported a phase II trial of modified FOLFIRINOX (mFOLFIRINOX) to evaluate its tolerability and efficacy [6], which involved a dose reduction of irinotecan to 150 mg/m^2^ and no intravenous bolus injection of 5-FU. Herein, the incidences of grade 3–4 neutropenia and febrile neutropenia were less than those reported previously in the original FOLFIRINOX study in Japan [7]. The efficacy of the 150 mg/m^2^ irinotecan dose was also as high as that of the original FOLFIRINOX [median OS 11.2 months and objective response rate (ORR) 37.7%] [6]. Therefore, mFOLFIRINOX is now commonly used for the treatment of advanced PDAC.

Irinotecan is a prodrug; its active metabolite (SN-38) shows the antitumor activity as well as toxicity. The enzyme UDP-glucuronosyltransferase 1 family polypeptide A1 (UGT1A1), encoded by *UGT1A1*, glucuronidates SN-38 to SN-38-G (an inactive metabolite). *UGT1A1* has germline polymorphisms such as UGT1A1*28 and UGT1A1*6. The wild-type allele (UGT1A1*1) has six TA repeats in the promoter region but the variant allele (UGT1A1*28) has seven TA repeats. Another variant allele (UGT1A1*6) consists of a single nucleotide replacement in exon 1 of the *UGT1A1* gene. UGT1A1*6 is important because it is one of the major polymorphisms among the Asian population but is rarely found in Caucasians [8]. Patients with genetic polymorphisms in *UGT1A1* have a dose-dependent increase in the risk of grade 3–4 hematologic toxicity and diarrhea [8–10]. Patients homozygous for UGT1A1*28 (UGT1A1*28/*28), homozygous for UGT1A1*6 (UGT1A1*6/*6), and heterozygous for both UGT1A1*28 and UGT1A1*6 (UGT1A1*6/*28) are defined as having double-variant-type (DV) UGT1A1 polymorphisms. In Japan, the frequency of DV is approximately 10%, comparable to that of UGT1A1*28 homozygotes in Caucasian populations [11, 12].

A dose reduction for DV patients with PDAC should be considered but the recommended initial dose of mFOLFIRINOX remains undetermined. Minami et al. have suggested that the dose of irinotecan for DV patients should be reduced to half of that recommended for *non*-DV patients. This is considering a 2.4-fold steep relationship between the dose of irinotecan and the AUC of SN-38 for DV patients compared with *non*-DV patients [12]. Sharma et al. have reported that the reduction of irinotecan dose to 90 mg/m^2^ in mFOLFIRINOX (oxaliplatin 85 mg/m^2^, irinotecan 90 mg/m^2^, leucovorin 400 mg/m^2^, and 5-FU 2400 mg/m^2^ over 46 h) was intolerable, and thus not feasible for patients with the UGT1A1*28/*28 genotype [13].

Considering these results, we hypothesized that further dose reduction of irinotecan was needed for DV patients. In this study, we, therefore, examined the effects of a reduced irinotecan dose of 80 mg/m^2^ in clinical practice for retrospectively evaluating the safety and efficacy of mFOLFIRINOX.

## Patients and methods

### Patients

**Fig. 1:**
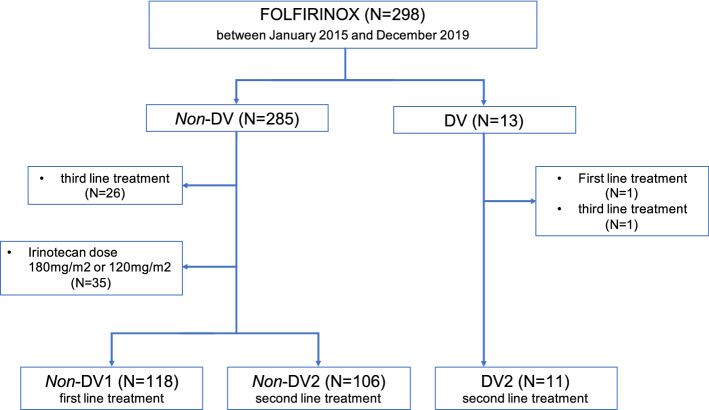
Study flow diagram

The subjects were patients with unresectable or recurrent pancreatic cancer who received FOLFIRINOX therapy at the National Cancer Center Hospital (NCCH) and were screened for *UGT1A1* genetic polymorphisms between January 2015 and December 2019. All subjects underwent a *UGT1A1* polymorphism test during diagnosis. We divided the patients into three groups according to *UGT1A1* polymorphism and treatment line. Patients with the DV possessed the UGT1A1*6/*6, UGT1A1*28/*28, and UGT1A1*6/*28 genotypes and received mFOLFIRINOX as second-line therapy (DV2). The other patients without DV received mFOLFIRINOX as first-line (*non*-DV1) or second-line therapy (*non*-DV2). Patients with DV treated with mFOLFIRINOX as first-line therapy were excluded because there was only one patient in this group (Fig. 1).

## Methods

### Treatment

DV patients received reduced dose of mFOLFIRINOX (every 2 weeks: oxaliplatin 85 mg/m^2^, irinotecan 80 mg/m^2^, leucovorin 200 mg/m^2^, and 5-FU 2400 mg/m^2^ over 46 h). Although this was not a prospective study, considering the previous reports of Minami et al. and Sharma et al., irinotecan dose was reduced to 80 mg/m^2^ for all-DV patients who chose mFOLFIRINOX as second-line therapy during the study period at our institute. We explained our strategy of treatment to the DV patients using a reduced dose of mFOLFIRINOX; a few patients wanted mFOLFIRINOX treatment without the reduced dose of irinotecan. *Non*-DV patients received the standard mFOLFIRINOX (every two weeks: oxaliplatin 85 mg/m^2^, irinotecan 150 mg/m^2^, leucovorin 200 mg/m^2^, and 5-FU 2400 mg/m^2^ over 46 h). *Non*-DV patients who had received mFOLFIRINOX with an initial dose of irinotecan (180 or 120 mg/m^2^) were excluded from this study (Fig. 1). Prophylactic filgrastim or pegfilgrastim in cycle 1 was not permitted.

### Assessment

This was a retrospective, single-center study conducted in NCCH and approved by the institutional review boards of the NCCH. Clinical data of the patients were obtained from the electronic medical records. The collected clinical data included age; sex; Eastern Cooperative Oncology Group performance status (ECOG-PS); extent of PDAC disease (recurrence, locally advanced, metastatic); an initial dose of irinotecan; first and last administration date of mFOLFIRINOX; type and severity of adverse events; and date of death [14]. We evaluated the safety and efficacy of irinotecan at a dose of 80 mg/m^2^ in the DV2 group in comparison with those of the *non*-DV1 (safety) or *non*-DV2 (safety and efficacy) groups. Regarding safety, grade ≥ 3 adverse events during the entire cycle of mFOLFIRINOX in the DV2 group were recorded according to the National Cancer Institute’s Common Terminology Criteria for Adverse Events v5.0 (CTCAE 5.0) and compared with those in *non*-DV1 and *non*-DV2 groups [15]. As for efficacy, we focused on second-line treatment cases, because we had only one case of first-line treatment in all of the DV patients and excluded it from the study. We, therefore, assessed the responses of progression-free survival (PFS) and overall survival only in second-line treatment cases. Tumor response was analyzed using the Response Evaluation Criteria in Solid Tumors (RECIST) version 1.1 [16]. The proportion of patients showing response was calculated as the total proportion of patients with a best overall response, complete or partial; the proportion of patients with disease control was calculated as the total proportion of patients with a best overall response, complete or partial, or with stable disease. Survival duration and occurrence frequencies of adverse events were analyzed up to February 2022. At this cutoff time, one patient in the DV2 group and five in the *non*-DV2 group were alive. The median length of follow-up was 10.5 months (range, 0.3–60.5) for all subjects.

### Statistical analysis

PFS was defined as the period from the start of second-line mFOLFIRINOX treatment to tumor progression or death. OS was defined as the period from the start of second-line mFOLFIRINOX treatment to death. Categorical variables were compared using Fisher’s exact test. The median values of the variables were compared using the Mann–Whitney *U* test. Time-to-event data were analyzed using standard methods, including Kaplan–Meier product-limit estimates, and compared between independent groups using the log-rank test. Statistical significance was set at *P* < 0.05. Statistical analysis was performed using EZR software version 1.38 (Saitama Medical Center, Jichi Medical University, Saitama, Japan) [17].

## Results

### Patient characteristics

A total of 235 patients were eligible for this study consisting of 224 patients with *non*-DV PDAC and 11 patients with DV PDAC. In the *non*-DV group, 118 patients (49.7%) received mFOLFIRINOX as first-line (*non*-DV1), and 106 patients (41.3%) as second-line (*non*-DV2) (Fig. 1) therapies. The patient characteristics are shown in Table 1. The median age at the initiation of mFOLFIRINOX was 62 years in the *non*-DV1, 62 years in the *non*-DV2, and 64 years in the DV2 groups. The proportion of female patients was higher in the DV group than in the *non*-DV group. The ECOG-PS score was 0 or 1 in almost all patients. Regarding the extent of disease, 66 patients (55.9%) in the *non*-DV1, 60 patients (56.6%) in *non*-DV2, and 5 patients (45.5%) in the DV2 groups had metastatic disease. In contrast, 42 patients (35.6%) in the *non*-DV1, 28 patients (26.4%) in the *non*-DV2, and 5 patients (45.5%) in the DV2 groups had locally advanced disease, while 10 patients (8.5%) in the *non*-DV1, 18 patients (17.0%) in the *non*-DV2, and 1 patient (9.1%) in the DV2 groups had a postoperative recurrence. In the DV2 group, the genotypes of *UGT1A1* were *6/*6 in two patients (18.2%), *28/*28 in one patient (9.1%), and *6/*28 in eight patients (72.7%). Whereas the genotypes of *UGT1A1* were *1/*1 in 62 *non*-DV1 patients (52.5%) and 54 of *non*-DV2 patients (50.9%); *1/*6 in 34 *non*-DV1 patients (28.8%) and 30 of *non*-DV2 patients (28.3%); and *1/*28 in 22 *non*-DV1 patients (18.6%) and 22 of *non*-DV2 patients (20.8%). The initial oxaliplatin dose was reduced only for two patients in the *non*-DV2 group, whereas in the *non*-DV1 and DV2 groups, dosages were not reduced. The initial 5-FU dose was not reduced for any patient. All patients in the DV2 group and 100 patients (94.3%) in the *non*-DV2 group received the first-line treatment with gemcitabine plus nab-paclitaxel. Other regimens for first-line treatment are shown in Table 1. The median number of mFOLFIRINOX treatment cycles was 13 in the *non*-DV1, 9 in the *non*-DV2, and 12 in the DV2 group.

**Table 1:** Patient characteristics

	*Non*-DV1 (*n* = 118) *n* (%)		*Non*-DV2 (*n* = 106) *n* (%)		DV2 (*n* = 11) *n* (%)	
Age (median, [range])	62 [24–75]		62 [38–75]		64 [47–74]	
Sex						
Male	71	(60.2)	62	(58.5)	1	(9.1)
Female	47	(39.8)	44	(41.5)	10	(90.9)
ECOG-PS						
0	66	(55.9)	57	(53.8)	6	(54.5)
1	52	(44.1)	48	(45.3)	5	(45.5)
2	0	(0.0)	1	(0.9)	0	(0.0)
Extent of disease						
Recurrence	10	(8.5)	18	(17.0)	1	(9.1)
Locally advanced	42	(35.6)	28	(26.4)	5	(45.5)
Metastatic	66	(55.9)	60	(56.6)	5	(45.5)
*UGT1A1*						
*1/*1	62	(52.5)	54	(50.9)	0	(0.0)
*1/*6	34	(28.8)	30	(28.3)	0	(0.0)
*1/*28	22	(18.6)	22	(20.8)	0	(0.0)
*6/*6	0	(0.0)	0	(0.0)	2	(18.2)
*28/*28	0	(0.0)	0	(0.0)	1	(9.1)
*6/*28	0	(0.0)	0	(0.0)	8	(72.7)
Irinotecan dose (mg/m^2^)						
150	118	(100.0)	106	(100.0)	0	(0.0)
80	0	(0.0)	0	(0.0)	11	(100.0)
Oxaliplatin dose (mg/m^2^)						
85	118	(100.0)	104	(98.1)	0	(0.0)
65	0	(0.0)	2	(1.9)	11	(100.0)
5-FU dose (mg/m^2^)						
2400	118	(100.0)	106	(100.0)	11	(100.0)
Number of mFOLFIRINOX treatment cycles (median, [range])	13 [1–59]		9 [1–65]		12 [1–36]	
1st line treatment						
GEM + nab-PTX	0	(0.0)	100	(94.3)	11	(100.0)
GEM + nab-PTX + other	0	(0.0)	2	(1.9)	0	(0.0)
GEM + S-1	0	(0.0)	1	(0.9)	0	(0.0)
GEM	0	(0.0)	3	(2.8)	0	(0.0)

### Adverse events

The grade 3–4 hematological and non-hematological adverse events reported in > 5% of patients are summarized in Table 2. In the *non*-DV1 and *non*-DV2 groups, major adverse events were neutropenia (33.9, 31.1%), febrile neutropenia (6.8, 3.8%), diarrhea (11.9, 2.8%), peripheral sensory neuropathy (9.3, 2.8%), and anorexia (7.6, 0.9%), respectively. In the DV2 group, neutropenia and febrile neutropenia were observed in 18.2% and 9.1% of the patients, respectively, and grade 3–4 adverse events, including diarrhea, peripheral sensory neuropathy, and anorexia, were not observed. Prophylactic filgrastim or pegfilgrastim was not used for all subjects. During mFOLFIRINOX treatment, filgrastim was administered to eight patients (6.8%) in the *non*-DV1, one (0.9%) in the *non*-DV2, and none in the DV2 group, as a treatment for febrile neutropenia. Interstitial lung disease (ILD) was found in one patient (0.8%) in the *non*-DV1, two (1.9%) in the *non*-DV2, and none in the DV2 groups. Although these ILD cases were of grade 1 or 2, mFOLFIRINOX treatment was discontinued in all three patients.

**Table 2:** Grade 3–4 hematological and non-hematological adverse events reported in > 5% of patients

	*Non*-DV1 (*n* = 118)				*Non*-DV2 (*n* = 106)				DV2 (*n* = 11)			
	Grade 3 *n* (%)		Grade 4 *n* (%)		Grade 3 *n* (%)		Grade 4 *n* (%)		Grade 3 *n* (%)		Grade 4 *n* (%)	
Adverse events												
Neutropenia	26	(22.0)	14	(11.9)	28	(26.4)	5	(4.7)	1	(9.1)	1	(9.1)
Febrile neutropenia	8	(6.8)	0	(0.0)	4	(3.8)	0	(0.0)	1	(9.1)	0	(0.0)
Diarrhea	14	(11.9)	0	(0.0)	3	(2.8)	0	(0.0)	0	(0.0)	0	(0.0)
Peripheral sensory neuropathy	11	(9.3)	0	(0.0)	3	(2.8)	0	(0.0)	0	(0.0)	0	(0.0)
Anorexia	9	(7.6)	0	(0.0)	1	(0.9)	0	(0.0)	0	(0.0)	0	(0.0)
Anemia	3	(2.5)	0	(0.0)	3	(2.8)	0	(0.0)	0	(0.0)	0	(0.0)
Thrombocytopenia	2	(1.7)	0	(0.0)	1	(0.9)	0	(0.0)	0	(0.0)	0	(0.0)

### Efficacy

**Fig. 2:**
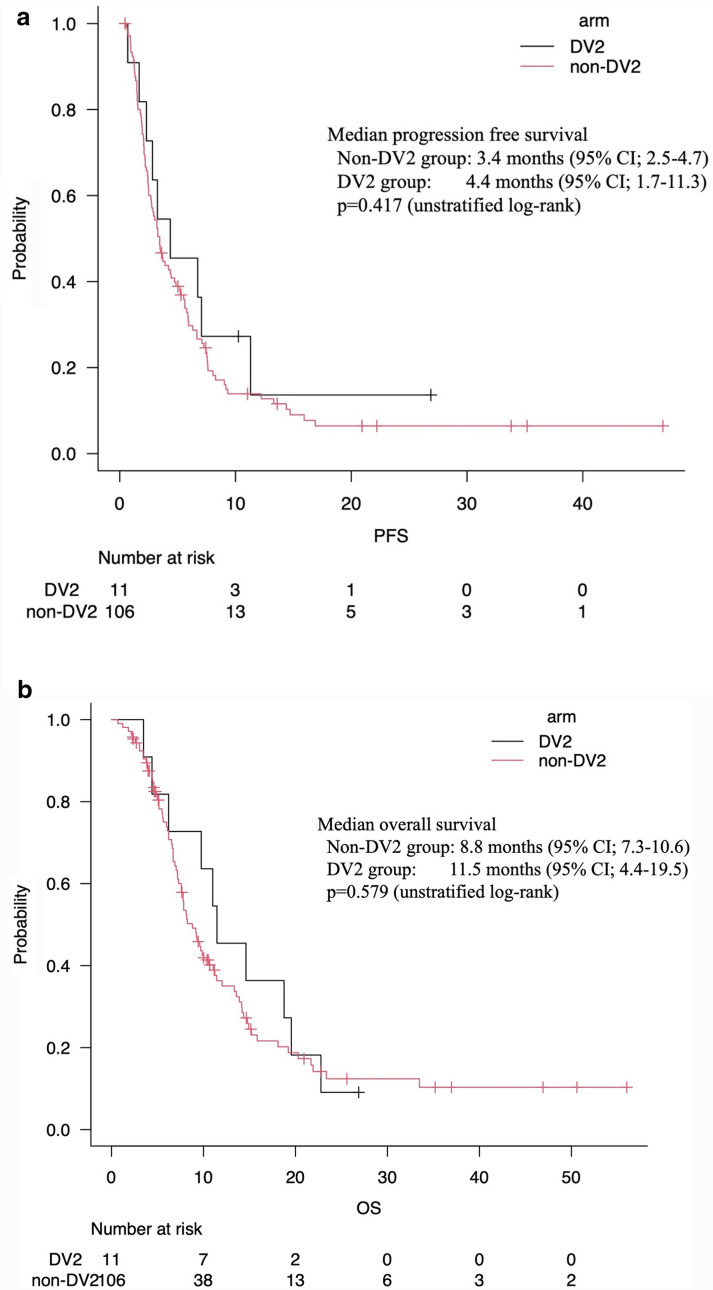
PFS **a** and OS **b** in patients treated with mFOLFIRINOX as second-line treatment. PFS and OS were calculated from the date of starting second-line chemotherapy. *PFS* Progression-free survival, *OS* overall survival

The treatment efficacy was evaluated using the best overall response based on the RECIST criteria and compared only in patients who received mFOLFIRINOX as second-line therapy. In the *non*-DV2 group, 1 patient (0.9%) had complete response, 13 patients (12.3%) had partial responses (PR), 39 patients (36.8%) had stable disease (SD), 52 patients (49.1%) had progressive disease (PD), and 1 patient (0.9%) was not evaluable for response. In the DV2 group, one patient (9.1%) had PR, six patients (54.5%) had SD, and four patients (36.4%) had PD. No significant difference in response rate (13.2% vs. 9.1%) or disease control rate (50.0% vs. 63.6%) was obtained between the *non*-DV2 and DV2 groups, respectively. The median PFS from the date of starting second-line chemotherapy was 3.4 months (95% CI 2.5–4.7) in the *non*-DV2 group and 4.4 months (95% CI 1.7–11.3) in the DV2 group. The median OS from the date of starting second-line chemotherapy was 8.8 months (95% CI 7.3–10.6) in the *non*-DV2 group and 11.5 months (95% CI 4.4–19.5) in the DV2 group. There was no significant difference in PFS (*P* = 0.417) or OS (*P* = 0.579) between *the non*-DV2 and DV2 groups (Fig. 2). In the *non*-DV1 group, 40 patients (33.9%) had PR, 54 patients (45.8%) had SD, 21 patients (17.8%) had PD, and 3 patients (0.9%) were not evaluated for response, while the disease control rate was 79.7%. The median PFS and OS from the date of starting first-line chemotherapy were 7.8 months (95% CI 5.9–10.1) and 16.2 months (95% CI 14.8–19.5).

## Discussion

FOLFIRINOX is a preferred treatment regimen for PDAC recommended in the NCCN guidelines [18]. However, it induces high-grade toxicity, such as myelosuppression, GI toxicity, and neuropathy. Therefore, mFOLFIRINOX, less toxic than the original FOLFIRINOX, is also a preferred regimen recommended in the NCCN guidelines and is commonly used worldwide. Although the *UGT1A1* polymorphism should be taken into consideration when choosing an irinotecan dose in treatments such as FOLFIRINOX, there is currently a paucity of evidence for a preferable dose reduction.

The safety of FOLFIRINOX in PDAC patients with wild and heterozygous UGT1A1*6 and *28 polymorphisms was assessed in Japanese patients by Shirasu et al. [19]. There was no difference in the frequency of adverse events depending on the *UGT1A1* status in patients with wild and heterozygous genotypes. Umemoto et al. suggested that an initial dose of irinotecan ≤ 120 mg/m^2^ is possibly the optimal dose for the first cycle of FOLFIRINOX for Japanese DV patients with advanced PDAC [20]. In this study, 11 patients were administered the 90–100 mg/m^2^ irinotecan dose. Within this subgroup, grade 4 neutropenia was observed in 27% of patients, and grade 3–4 adverse events, such as febrile neutropenia, diarrhea, and anorexia, were observed in 18% of patients. These results were negative for the safety of FOLFIRINOX with reduced irinotecan dose (90–100 mg/m^2^). In another study, Sharma et al. reported that dose-limiting toxicity occurred in 4/10 (40%) in DV (UGT1A1*28/*28) patients who received mFOLFIRINOX with a 90 mg/m^2^ irinotecan dose, and this dose reduction was not tolerable for DV patients with pancreatic and biliary tract cancers [13]. Minami et al. suggested that the dose of irinotecan for DV patients should be reduced to half of the dosage recommended for other patients, considering a 2.4-fold steep relationship between the dose of irinotecan and the AUC of SN-38 for DV patients compared with *non*-DV patients [12]. Based on these results, we hypothesized that further dose reduction of irinotecan is needed for DV patients with PDAC. Consequently, our study showed a milder toxicity profile than those previously reported for DV patients and a Japanese phase II study for *non*-DV patients (Table 3) [6, 13, 20]. These results from our study indicated that mFOLFIRINOX with a reduced dose of irinotecan at 80 mg/m^2^ is tolerable for DV2 patients. Moreover, *non*-DV1 patients had slightly more grade 3–4 adverse events than *non*-DV2 patients. This result may indicate a selection bias. *Non*-DV2 and DV2 patients in our study were not treated with milder chemotherapy such as nanoliposomal irinotecan in combination with 5-FU and folinic acid or S-1 monotherapy, but by mFOLFIRINOX as second-line therapy. These *non*-DV2 and DV2 patients might be in better general condition than *non*-DV1 patients. This bias could also occur in DV patients; therefore, if we use mFOLFIRINOX as first-line therapy for DV patients, more attention should be paid to toxicity. As for febrile neutropenia (FN), granulocyte colony-stimulating growth factor (G-CSF) decreases the risk of FN in patients receiving myelosuppressive chemotherapies. A recent systematic review showed that prophylactic use of G-CSF reduces the rates of FN, dose reduction, and treatment delay [21]. Our group previously conducted a phase II trial to evaluate the safety and efficacy of primary prophylactic pegfilgrastim in patients with metastatic pancreatic cancer who received FOLFIRINOX [22]. However, FN occurred in 18.0% and previously, we could not demonstrate the efficacy of pegfilgrastim addition to FOLFIRINOX; the DV patients were excluded. Thus, the efficacy of prophylactic pegfilgrastim in DV patients needed elucidation.

**Table 3:** Comparison of grade 3–4 adverse events, irinotecan dose, and *UGT1A1* polymorphism in this study with previous reports

Authors	*n*	UGT1A1	Irinotecan (mg/m^2^)	Neutropenia (%)	Febrile neutropenia (%)	Diarrhea (%)	Peripheral sensory neuropathy (%)	Anorexia (%)
Previous reports								
Sharma et al.	10	DV	90	NE	20	10	0	0
Umemoto et al.	31	DV	≤ 60–180	65	13	6	6	16
Ozaka et al.	69	Non-DV	150	47.8	8.7	10.1	5.8	15.9
This study								
*Non*-DV1	118	Non-DV	150	33.9	6.8	11.9	9.3	7.6
*Non*-DV2	106	Non-DV	150	31.1	3.8	2.8	2.8	0.9
DV2	11	DV	80	18.2	9.1	0	0	0

For efficacy, we assessed second-line treatment cases because we had only one case for first-line treatment in all-DV patients (excluded from the study). Along with FOLFIRINOX, gemcitabine + nab-paclitaxel (GnP) treatment is another preferred first-line treatment of PDAC. For patients treated with GnP or other gemcitabine-based regimens as first-line treatment, nanoliposomal irinotecan + 5-FU / leucovorin treatment as a second-line treatment showed preferable results in phase III (NAPOLI-1) study and became one of the recommended second-line treatment regimens. This NAPOLI-1 regimen achieved an ORR of 16%, a disease control rate of 52%, while median PFS was 3.1 months, and OS was 6.1 months [23]. FOLFIRINOX is another treatment option for second-line treatment after gemcitabine-related regimens, especially for patients with good PS. In Table 4, the results from major previous reports for second-line FOLFIRINOX are shown together with our study results [24–29]. In our study, there were no significant differences between the DV2 and *non*-DV2 groups, not only in treatment response and disease control but also in PFS and OS. Relative to previous findings, our DV2 group did not show a high response rate; however, the disease control rate was better. Although other study results could not be compared without considering patient characteristics, mFOLFIRINOX with a reduced dose of irinotecan at 80 mg/m^2^ likely showed as high efficacy as a previous study with FOLFIRINOX and NAPOLI-1 trials. As for first-line mFOLFIRINOX therapy, we showed strong results not only in safety but also in efficacy in *non*-DV1 patients.

**Table 4:** Summary of therapeutic effects of second-line FOLFIRINOX in this study and their comparison with previous reports

Authors	*n*	Treatment	Irinotecan (mg/m^2^)	ORR (%)	DCR (%)	Median PFS (months) [95% CI]	Median OS (months) [95% CI]
Previous reports							
Kobayashi et al.	44	FOLFIRINOX	180	25	59	4.1 [2.6–5.5]	10.3 [7.2–13.3]
Foschini et al.	15	FOLFIRINOX	180	46	78	22.3 (weeks) [10.7–65.3]	47.9 (weeks) [12.3–98.3]
Sawada et al.	104	mFOLFIRINOX	150	10.6	56.7	3.9 [2.8–5.0]	7.0 [6.2–9.8]
Kim et al.	39	mFOLFIRINOX	135	10.3	64.1	3.8 [1.5–6.0]	8.5 [5.6–11.4]
Saito et al.	35	mFOLFIRINOX	150	2.7	62.2	5.7 [3.3–12.6]	11.5 [7.1–14.5]
Chung et al.	48	mFOLFIRINOX	120	18.8	62.5	5.8 [3.7–7.9]	9.0 [6.4–11.6]
This study							
*Non*-DV2	106	mFOLFIRINOX	150	13.2	50.0	3.4 [2.7–4.6]	8.8 [7.6–11.1]
DV2	11	mFOLFIRINOX	80	9.1	63.6	4.4 [1.7–11.3]	11.5 [4.9–19.5]

To the best of our knowledge, this study is the first to report the safety and efficacy of mFOLFIRINOX with a fixed, reduced dose of irinotecan (80 mg/m^2^) for DV patients. In addition, our results showed the safety and efficacy of second-line mFOLFIRINOX in a large number of patients, including both DV and *non*-DV, and we have discussed how our results were comparable with previous results. However, this study had some limitations. It was a single-center retrospectively conducted study, and the sample size was small. The *UTG1A1* genotype difference in DV patients could have some effects on safety and efficacy. In our study, the genotypes of *UGT1A1-DV* were almost all *6/*28 double heterozygous type, and only a few patients had *6/*6 (two patients) or *28/*28 homozygous type (one patient). This proportion in the *UGT1A1* genotype differed from those in the Umemoto and Sharma studies [13, 20]. As for efficacy, we evaluated only second-line treatment cases. In our institute, most DV patients received GnP or other gemcitabine-related regimens for first-line treatment, because we have limited data about the safety and efficacy of mFOLFIRINOX as first-line treatment in DV patients.

In conclusion, the results of our study indicated that 80 mg/m^2^ irinotecan mFOLFIRINOX was relatively well tolerated, suggesting that it may serve as a second-line treatment option for DV patients with PDAC. Our results also demonstrated relatively better disease control efficacy as second-line therapy. Further studies are necessary to evaluate the safety and efficacy of first-line treatment for DV patients with PDAC.
